# Free-Floating Scapular Spine: A Rare Shoulder Injury

**DOI:** 10.1155/2019/1839375

**Published:** 2019-09-30

**Authors:** John H. Cabot, Khang H. Dang, Anil K. Dutta

**Affiliations:** Department of Orthopaedics, UT Health San Antonio, San Antonio, TX 78229, USA

## Abstract

A specific treatment protocol for managing scapular spine fractures does not currently exist. The purpose of our report is to describe this type of injury and detail our treatment management in order to better elucidate this rare pathology. We present a case of a 26-year-old female with an acute scapular spine and base fracture after a motor vehicle collision. Successful treatment of an acute free-floating scapular spine fracture was achieved with open reduction and internal fixation utilizing an elbow plate. Since scapular spine fractures are an unfortunate, rare injury, it may impose difficult challenges to the treating surgeon. With our case report, we hope to contribute to the overall knowledge of scapular spine fractures and offer our experience with a successful and appropriate treatment option in our patient.

## 1. Introduction

Acute scapula fractures are a rare injury accounting for 3-5% of fractures of the shoulder girdle and occurring at a rate of 0.7% for all fractures [1]. These injuries are generally a result of high-energy mechanisms, such as motor vehicle collisions, and are often associated with other upper extremity, thoracic, or pelvic ring injuries [2]. The most common scapula fractures involve the neck or the body, which represents two-thirds of all scapula fractures [3]. Conversely, scapular spine fractures are a rare subset accounting for only 6% of all scapula fractures and generally healed with conservative management [4, 5]. We present a case of a 26-year-old female with a fracture of the scapular body and spine with extension into the base of the acromion. Our case may shed light on what we believe is the first documented case report of free-floating scapular spine fracture, with consideration of a potential treatment option.

## 2. Case Report

**Figure 1:**
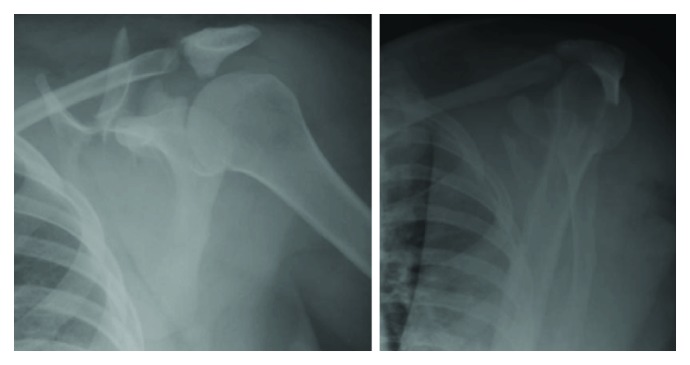
AP and lateral radiographic views of the scapula showing a comminuted and displaced fracture of the scapular spine and body with an avulsion of the acromion base.

**Figure 2:**
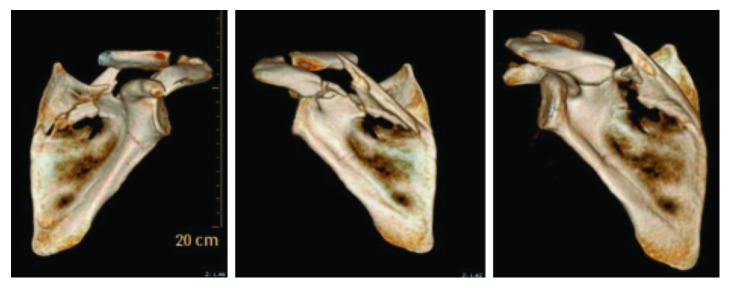
Computed tomography 3-dimensional reconstruction of the scapula showing a segmental fracture of the entire superior aspect including the acromion along with free-floating fragments of its base and spine.

A 26-year-old right-handed female with no pertinent medical history presented to our emergency department with left shoulder pain after a motor vehicle collision in early 2019. The patient was a restrained passenger in a high-speed motor vehicle rollover two days before her presentation, resulting in a fatality at the scene. She was initially seen at an outside facility who diagnosed her with a clavicle fracture and multiple rib fractures. Her physical exam showed no neurovascular deficits but significant pain and swelling over the scapula along with superficial abrasions. Radiographs showed a comminuted and displaced fracture of the scapular spine and body with an avulsion of the acromion base (Figure 1). Computed tomography 3-dimensional reconstruction revealed no glenoid involvement but a segmental fracture of the entire superior aspect of the scapula, including the acromion base and spine, which were free-floating fragments (Figure 2). Given the degree of displacement and increased risk of nonunion and poor functional outcome, the patient elected to proceed with open reduction and internal fixation one week following her injury.

**Figure 3:**
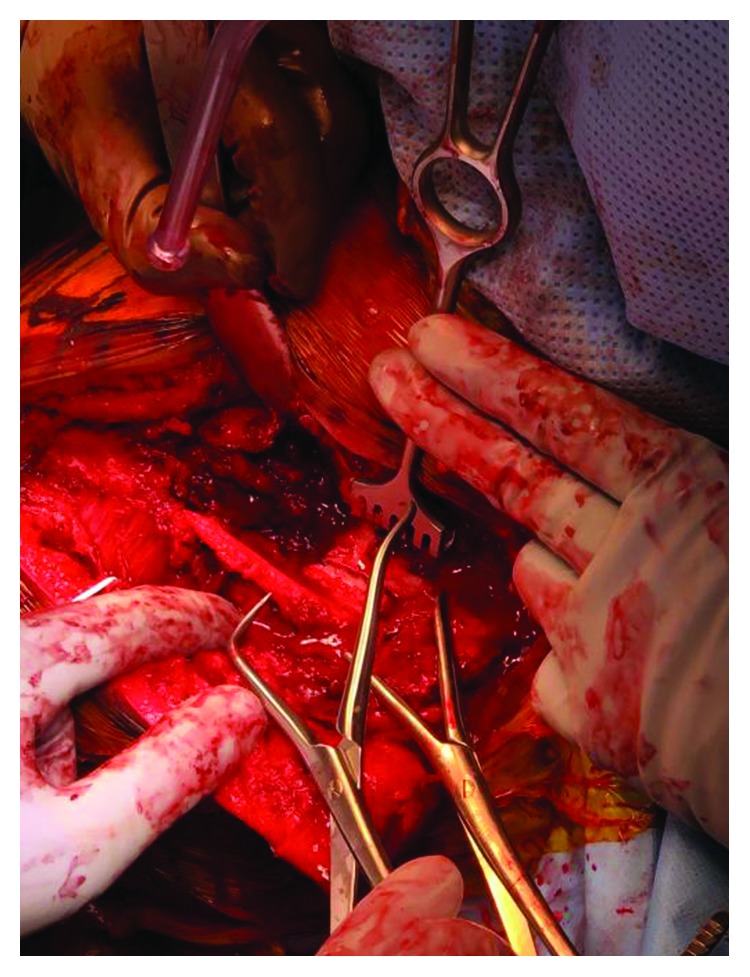
Intraoperative view of the temporary reduction of scapular spine fracture with forceps.

**Figure 4:**
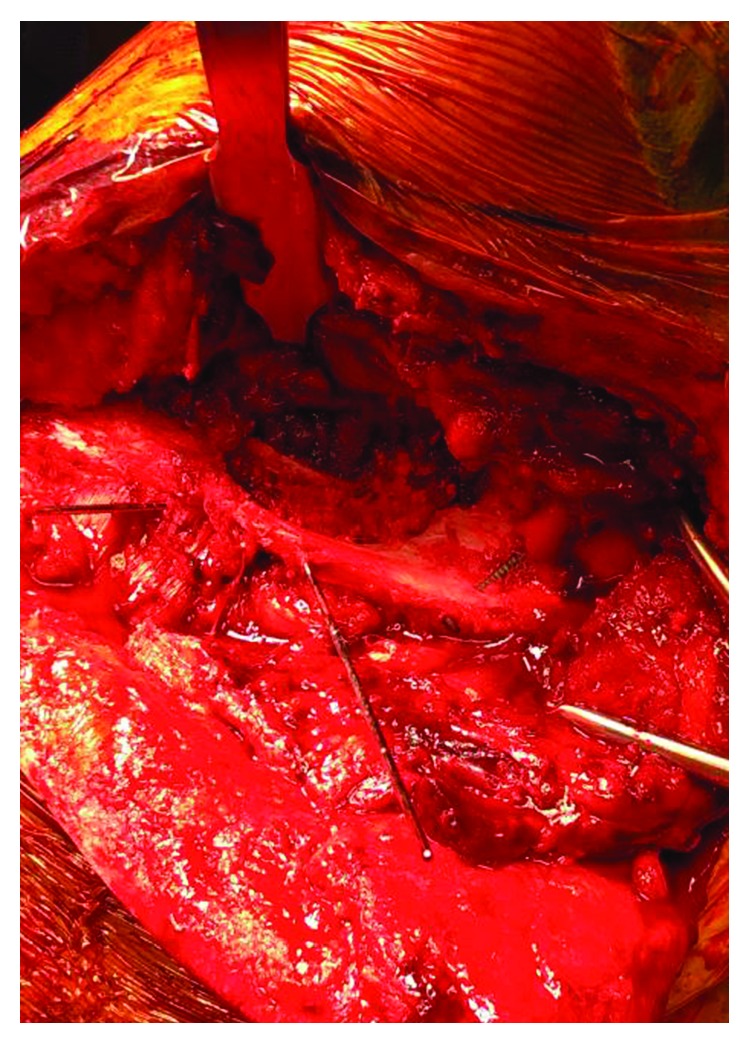
2.7 mm lag screws and K-wires were used to provide temporary fixation and alignment of the scapular spine fracture.

**Figure 5:**
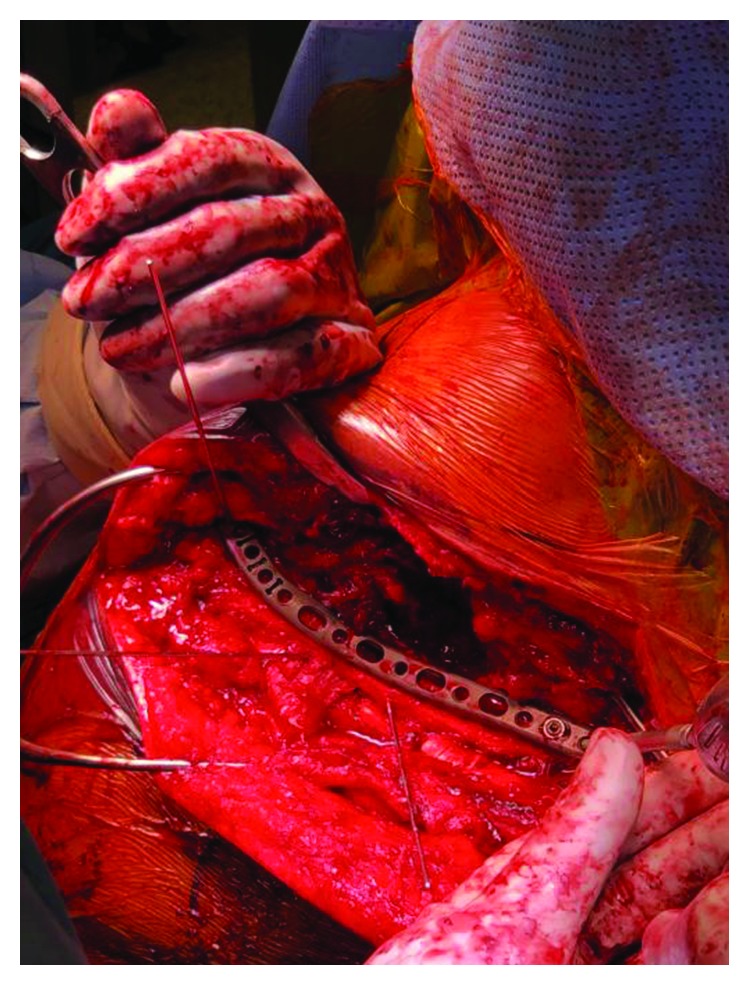
Intraoperative view of the placement of the Zimmer-Biomet elbow plate which matched the contour of the spine of the scapula.

**Figure 6:**
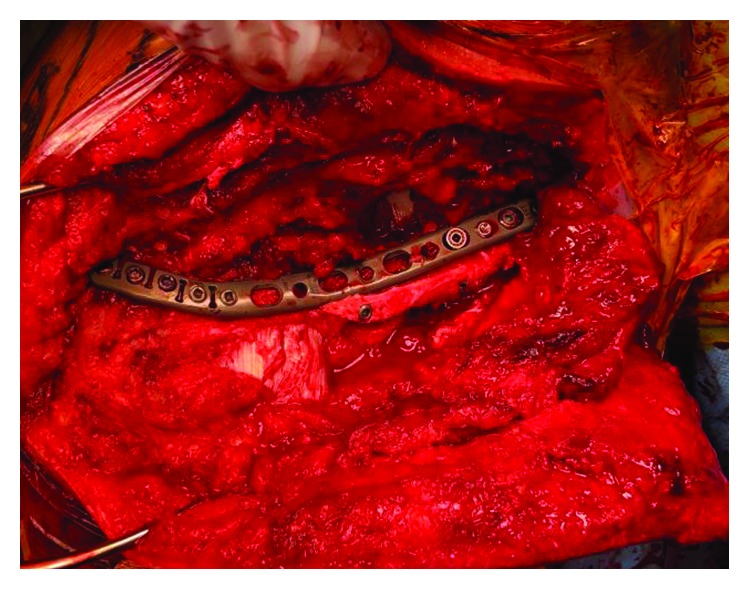
Intraoperative views showing our final plating construct which was supported with multiple cortical screws from superior and posterior to anterior and inferior.

Intraoperatively, the patient was positioned upright in the beach chair position. With an incision along the scapular spine, a posterior approach to the scapula was utilized. The trapezius muscle was elevated off the acromion and scapular spine while the posterior deltoid origin was maintained on the lateral aspect of the acromion. The supraspinatus and infraspinatus were elevated subperiosteally from their origin on the scapula towards their insertion thus allowing for full exposure of the entire superior body of the scapula. After adequate exposure, the posterior portion of the acromion was reduced to the scapular body with point of reduction clamps and stabilized with 2.7 mm lag screws (Figures 3 and 4). Temporary reduction of the fractured scapular spine to the acromion was achieved with K-wires, and then, a Zimmer Biomet elbow plate was placed for definitive fixation (Figure 5). The curvature of the elbow plate matched the curvature of the acromion, and we were able to place multiple cortical screws from superior and posterior to anterior and inferior (Figure 6). Our plating construct had excellent bony purchase and maintained reduction of the acromion to the scapular body.

**Figure 7:**
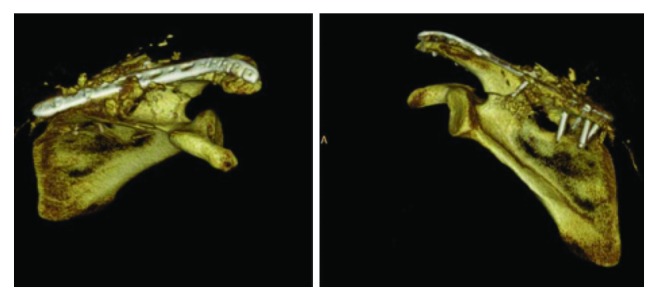
Postoperative computed tomography 3-dimensional reconstruction of the scapula showing our plate fixation of the scapular spine fracture.

**Figure 8:**
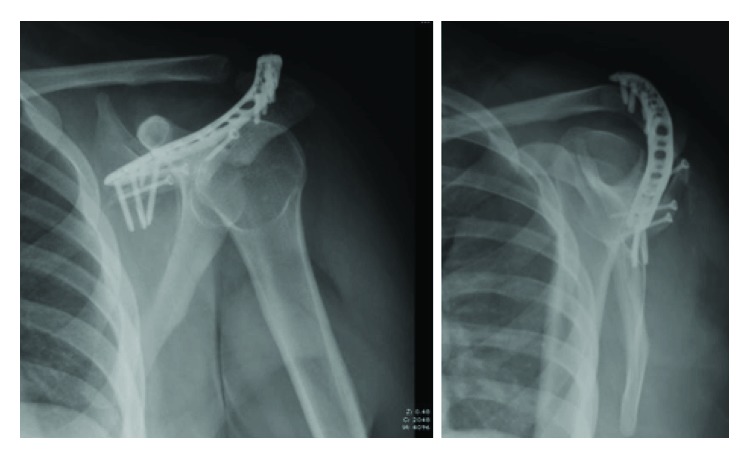
AP and lateral radiographic views of the scapula showing our fracture in appropriate alignment with evidence of implant failure or loosening.

The patient had no perioperative complications and was discharged from her hospital stay without issues. She was briefly immobilized for two weeks and then started shoulder range of motion training followed by strengthening. Unfortunately, she did not follow up after seven months due to moving away from our location. At her last follow-up, she reported mild pain in her scapula with motion but otherwise had no complaints or neurovascular deficits. Regarding her function, she lacked terminal motion in all directions and has mild weakness compared to her contralateral side. However, she returned to all her daily activities without significant limitation to her sporting activities. Her last radiographic assessment showed maintained alignment of the acromion process without implant failure (Figures 7 and 8).

## 3. Discussion

Scapula fractures are an uncommon injury accounting for less than 1% of all fractures, with scapular spine accounting only 6% of these [4, 5]. The infrequency is due to the mobility of the scapula, allowing displacement of traumatic forces, as well as protection by the thoracic cavity anteriorly and the thick layer of soft tissue and muscles posteriorly [6]. Therefore, these fractures generally occur as a result of direct force from high-energy mechanisms such as motor vehicle collisions or autopedestrian accident. To the best of our knowledge, we present the first documented case of a free-floating scapular spine treated with our technique.

Scapular spines have a considerable amount of variability in their shape, thus adding to the difficulty of fixation. Wang et al. [7] developed a classification system of scapular spines, and according to this scheme, our patient's scapular spine fracture is classified as a Type IV or a “wooden club shape,” the third most common morphology. Of note, patients with Type V or Type I classification have a more complex morphology that may result in more difficult fixation [7]. However, the literature on acute scapular spine fractures has been scarce.

Previous literature reported on the spontaneous occurrence of these fractures in the elderly or chronic scapular spine fractures. In elderly patients with poor bone quality, atraumatic scapular spine fractures have been reported as a result of chronic rotator cuff tear arthropathy or following reverse total shoulder arthroplasty. These fractures are usually nondisplaced and are treated conservatively [8–12]. Another etiology of atraumatic fractures of the scapular spine is avulsion fractures resulting from voluntary muscle contraction of the deltoid. These fractures are incredibly uncommon; current literature supports both conservative and operative management of these fractures [13, 14]. As-Sultany et al. [15] reported a case of a minimally displaced scapular spine fracture in a 39-year-old male that failed nonoperative management and developed a nonunion of the spine at the base of the acromion. He subsequently underwent plate fixation along with autograft placement from his iliac crest and eventually returned to full functional status post five months postoperatively. The excellent outcome achieved through their surgical treatment led the authors to have a low threshold for operative fixation of displaced scapular spine fractures, especially in healthy, younger patients [15]. Overall, there are no extensive studies on fractures of the scapular spine and their outcomes from various treatment options.

Our treatment consisted of open reduction and internal fixation of the scapular spine, and we achieved adequate fixation and good functional outcome. We stress the importance of proper exposure since we were able to assess the full extent of the fracture pattern for our fixation. While a modified Judet approach, as described by Cole and Dugarte [16], can be used for less extensive injuries to decrease soft tissue trauma, we opted to use the extensile Judet approach to raise the posterior musculature to evaluate this complex fracture pattern fully. Once we exposed the fracture site, 2.7 mm lag screws were used to reduce the posterior portion of the acromion to the scapular body and had excellent bony purchase. This temporary fixation was stabilized further with K-wires to allow for our definitive fixation. Scapular plating systems are not common for the scapular spine, and we found that an elbow plate matched the contour of the scapular spine. Additionally, this plating construct allowed us to place several cortical screws from superior and posterior to anterior and inferior, thus bolstering our fixation.

## 4. Conclusion

While scapular spine fracture is an unfortunate, rare injury that may impose difficult challenges to the treating surgeon, treatment guidelines for the management of these fractures currently do not exist. Here, we detailed the successful treatment of an acute free-floating scapular spine fracture after open reduction and internal fixation with an elbow plate. With our case report, we hope to contribute to the overall knowledge of scapular spine fractures and offer our experience with a successful and appropriate treatment option in our patient.
